# Comparison of complications between laparoscopic and open abdominal approaches in morbidly obese patients with early-stage endometrial carcinoma

**DOI:** 10.2478/raon-2026-0021

**Published:** 2026-04-16

**Authors:** Ajda Kljajic, Branko Cvjeticanin, Borut Kobal, Matija Barbic, Spela Smrkolj, Ivan Verdenik, Mija Blaganje

**Affiliations:** 1 Department of Gynecology and Obstetrics, General hospital Slovenj Gradec, Slovenj Gradec, Slovenia; 2 Department of Gynecology and Obstetrics, University Medical Centre Ljubljana, Ljubljana, Slovenia; 3 Medical Faculty, University of Ljubljana, Ljubljana, Slovenia

**Keywords:** endometrial carcinoma, morbid obesity, laparoscopic hysterectomy, surgical outcomes, Clavien-Dindo Classification

## Abstract

**Background:**

Endometrial carcinoma is the most common gynecological malignancy globally. Its rising incidence is closely linked to the increasing prevalence of morbid obesity (body mass index [BMI] > 40 kg/m^2^), which elevates the technical difficulty of surgery and the risk of perioperative complications. Identifying the optimal surgical approach is critical for this high-risk population.

**Patients and methods:**

This retrospective study with prospectively collected data compared laparoscopic (LPSC) versus open abdominal (LAP) surgical approaches for low risk endometrioid carcinoma in morbidly obese patients. Data were collected over an eleven-year period (January 2013–December 2023) and included 73 patients (58 LPSC, 15 LAP) who met the inclusion criteria (BMI > 40 kg/m^2^, low-grade, early-stage endometrioid carcinoma). Outcomes measured included operative time, intraoperative blood loss, length of hospital stay, and intraoperative/postoperative complications, which were rigorously classified using the Clavien-Dindo system.

**Results:**

Baseline patient characteristics were comparable between the two groups. The LPSC group demonstrated significantly superior perioperative outcomes. The average postoperative hospital stay was markedly shorter in the LPSC group (4.5 days) compared to the LAP group (12.7 days). Furthermore, LPSC was associated with lower rates of reoperation, transfusions, and postoperative anemia. Crucially, LPSC resulted in a statistically lower occurrence of severe postoperative complications (Clavien-Dindo Grade II and III).

**Conclusions:**

he laparoscopic approach offers clear and significant perioperative advantages over open abdominal surgery for morbidly obese patients with low-risk endometrial carcinoma. Given the improved safety profile, LPSC or robotic-assisted surgery should be established as the preferred initial surgical approach in these technically challenging cases.

## Introduction

Endometrial cancer is the most common gynecological malignancy in Europe and worldwide. It is estimated that in 2018, 121,578 women in Europe were diagnosed with endometrial cancer, and 29,638 died from the disease. Its incidence increases with population aging and the rising proportion of overweight individuals.^1^

Endometrial carcinoma as the most frequent gynecological malignancy among Slovenian women ranks fifth among all cancers in terms of incidence. Between 2016 and 2020, an average of 367 women per year were diagnosed with endometrial cancer in Slovenia, and 71 women died annually. During this period, the incidence rate was 35.2 per 100,000.^2^

One of the significant risk factors for the development of endometrial carcinoma is excessive body weight. It is estimated that more than half of endometrial carcinomas are associated with obesity.^3^

In particular, morbid obesity, which represents an increasing global health problem and is defined by the World Health Organization as a body mass index (BMI) greater than 40 kg/m^2^ confers up to a nine-fold higher risk of developing endometrial cancer compared to women with normal body weight (BMI < 25 kg/m^2^).^4,5–7^ According to various studies, between 19% and 36% of women with endometrial carcinoma are morbidly obese. These patients are technically more challenging to operate on and frequently present with obesity-related comorbidities. This leads to a higher risk of perioperative complications.^3,5,8^

In recent years, several studies focusing on morbidly obese patients have suggested that a laparoscopic approach to endometrial cancer surgery is preferable to open abdominal surgery (laparotomy). Laparoscopy has been associated with fewer perioperative complications and shorter hospital stays.^9–12^

## Patients and methods

Our study, which compared laparoscopic and open abdominal surgical approaches, was designed as a retrospective study with prospectively collected data. Ethical clearance for this retrospective study was obtained from the Institutional Ethics Committee of University Medical Centre Ljubljana, ethical approval KSEV-3-221024.

Inclusion criteria were:

- Age over 18 years;
- BMI greater than 40 kg/m^2^;
- preoperatively histologically confirmed low-grade endometrioid carcinoma with less than 50% myometrial invasion;
- no evidence of disease spread.

Patients were excluded if they had non-endometrioid histology, advanced endometrial carcinoma with evidence of invasion or dissemination, or if they had undergone surgery via the vaginal approach.

As recommended by international guidelines, in patients with low-risk endometrial carcinoma included in this study, treatment consisted of hysterectomy with bilateral adnexectomy and sentinel lymph node biopsy or pelvic lymphadenectomy. The choice of surgical approach was left to the surgeon’s discretion.

We compared the two groups (laparoscopic vs. open abdominal approach) in terms of baseline patient characteristics, operative time, intraoperative blood loss, length of hospital stays, intraoperative and postoperative complications, and the need for transfusion.

Surgical complications were classified according to the Clavien-Dindo classification:^13^

*Grade I*: any deviation from the normal postoperative course without the need for pharmacological treatment, surgical, endoscopic, or radiological intervention. Allowed treatments include antiemetics, antipyretics, analgesics, diuretics, electrolytes, and physiotherapy.

*Grade II*: complications requiring pharmacological treatment other than those permitted in Grade I. This also includes blood transfusions and total parenteral nutrition.

*Grade III*: complications requiring surgical, endoscopic, or radiological intervention.

*Grade IV:* life-threatening complications requiring ICU management due to single- or multi-organ dysfunction.

*Grade V*: patient death.

Statistical analysis was performed using IBM SPSS Statistics. The Mann-Whitney U test was used for comparisons of continuous variables, while the χ^2^ test and Fisher’s exact test (for small sample sizes) were used for categorical variables.

## Results

Data were collected over an eleven-year period from January 2013 to December 2023. Approximately 50% of all patients with endometrial carcinoma in our country are surgically treated at out department. Patients are referred for operative management through the national Gynecological Oncology Multidisciplinary Board, ensuring centralized decision making and strict adherence to standardized treatment pathways.During the study period 91 patients meeting the inclusion criteria underwent surgery at the Department of Gynecology. Of these, 58 patients underwent a laparoscopic approach (LPSC), while 15 patients were operated on with an open abdominal approach (LAP). In six patients initially assigned to the LAP group, surgery was started laparoscopically but subsequently converted to an open procedure due to extensive pelvic adhesions identified during laparoscopy, which significantly altered normal pelvic anatomy.

Patient age, BMI, and prevalence of comorbidities were comparable between the two groups (Table 1).

**TABLE 1.** Comparison of laparoscopic (LPSC) and open abdominal surgical approaches (LAP) groups by age, body mass index (BMI), and comorbidities

	LPSC N = 58	LAP N = 15	p-value
**Age, mean (SD)**	59 (9.3)	61 (9.6)	0.503
**BMI, mean (SD)**	45.8 (5.6)	46.1 (5.3)	0.924
**Percentage of patients with associated comorbidities**	87.9%	86.7%	0.594

Histological examination confirmed endometrioid endometrial carcinoma in all patients. In the LPSC group, 54 patients (93.1%) were classified postoperatively as stage IA, 2 (3.4%) as stage IB, and one patient (1.7%) each as stage II and stage III. In the LAP group, 11 patients (73.3%) were classified as stage IA, 2 (13.3%) as stage IB, and 2 (13.3%) as stage II.

We compared the frequency of intraoperative and postoperative complications between the two groups. The study focused on the occurrence of injuries to the urinary tract, bowel, and blood vessels. In the laparoscopic group, we recorded one case of bowel injury. Postoperatively, the patient was diagnosed with a rectovaginal fistula, which required reoperation on the eleventh postoperative day via open abdominal approach, including a protective ileostomy and fistula repair.

We also examined the proportion (53%) of wound-related complications after surgery in the group of patients operated on by open abdominal approach. There were eight wound-related complications, including 3 cases of wound dehiscence. Wound-related complications were not observed in laparoscopic group.

For patients in both groups, we calculated the average intraoperative blood loss, the proportion of blood transfusions, and the proportion of moderate anemia (defined as a hemoglobin level below 110 g/L) after surgery. We also recorded the rates of reoperation, operative time, and length of hospital stay after the procedure. The comparison of these complications between the two groups is presented in Table 2.

**TABLE 2.** Comparison of complications between the laparoscopic (LPSC) and open abdominal surgical approaches (LAP) groups

	LPSC N = 58	LAP N = 15	p-value
**Urinary tract injury (N)**	0	0	/
**Bowel injury (N)**	1	0	0.792
**Vascular injury (N)**	1	0	0.795
**Blood loss (ml), mean, (range)**	250 (50–1000)	300 (150–500)	0.241
**Percentage of transfusions**	5.2%	20.0%	0.097
**Percentage of moderate anemia**	22.4%	66.7%	0.002
**Operative time (min), mean, (range)**	106 (45–163)	119 (65–195)	0.353
**Percentage of revisions**	3.4%	20.0%	0.024
**Post-operative hospitalization time (days), mean, (range)**	4.5 (2–47)	12.7 (5–55)	< 0.001

Complications arising from the surgical procedure were also classified according to the Clavien-Dindo classification.^13^

The groups of patients who experienced a normal postoperative course and those classified as Grade I according to the Clavien-Dindo classification were presented together in the table. The table also shows the comparison of the number of patients classified as Grade II, III, and IV according to the classification above. No cases of patient death, corresponding to Grade V in the Clavien-Dindo classification, were observed in our cohort (Table 3,4).

**TABLE 3.** Comparison of complications using the Clavien-Dindo classification

Clavien-Dindo Classification	LPSC (N)	LPSC (%)	LAP (N(	LAP (%)
**Normal postoperative course or Grade I**	39	67.2	0	0
**Grade II**	17	29.3	12	80.0
**Grade III**	0	0	3	20.0
**Grade IV**	2	3.4	0	0
**Grade V**	0	0	0	0

1 LAP = open abdominal surgical approach group; LPSC = laparoscopic approach group

**TABLE 4.** Analysis of postoperative complications (Clavien-Dindo classification) according to surgical approach.

Statistical test	Value	p-value
Pearson Chi-square	29.912	< 0.001
Likelihood Ratio	34.818	< 0.001

## Discussion

Over eleven years, 58 morbidly obese patients (79.4%) at the Department of Gynecology underwent surgery via a laparoscopic approach, while 15 morbidly obese patients (20.6%) were operated on using an open abdominal approach.

In the laparoscopic group, we observed a significantly shorter hospital stay, a lower rate of reoperations, fewer transfusions, and postoperative anemia. Classification of postoperative complications according to the Clavien-Dindo system revealed a higher occurrence of Grade II and III complications in the group of patients operated on via the open abdominal approach.

Over the past two decades, numerous retrospective studies have evaluated the surgical management of endometrial carcinoma. While the LACC trial raised significant concerns regarding the oncologic safety of minimally invasive surgery in early-stage cervical cancer, those findings are disease-specific and have not been replicated in endometrial cancer research.^14^ Conversely, randomized trials and large prospective studies in endometrial carcinoma have consistently demonstrated that minimally invasive surgery offers equivalent oncologic outcomes alongside significantly reduced perioperative morbidity. Specifically, laparoscopic and laparoscopically assisted vaginal hysterectomy, compared to open abdominal hysterectomy, are associated with a significantly lower number of surgical complications, including postoperative pain, blood loss, the need for transfusion, and length of hospital stay.^15–18^

In recent years, due to the increasing population of morbidly obese patients and the well-documented elevated risk of endometrial carcinoma in this population, more studies have focused on comparing laparoscopic versus open abdominal surgical approaches specifically in morbidly obese patients.^19^ Our analysis shows similar results within our cohort.

In the laparoscopic group, we observed a statistically significant shorter postoperative hospital stay, which has also been reported in numerous other studies. After laparoscopic surgery, patients in our institution were hospitalized for an average of 4.5 days, comparable to other studies reporting 1.5 to 6 days. In the group of patients operated on via open abdominal approach, the average hospital stay was 12.7 days, while in other studies it ranged from 4 to 10 days.^5,10,20–22^ Estimated intraoperative blood loss was similar between the groups. However, based on the proportion of transfusions and the rate of moderate postoperative anemia, it is likely that greater blood loss occurred during open abdominal surgeries. Eltabbakh *et al*. made similar observations and concluded that intraoperative blood loss during laparoscopic surgery is often underestimated, as repeated irrigation and suction make accurate assessment more challenging.^20^ Several other studies have also reported lower blood loss during laparoscopic procedures.^10,21–23^

Given that open abdominal surgery is associated with a higher risk of wound-related complications (dehiscence, infection, etc.), we specifically focused on their incidence. As observed by Cheng and colleagues, our study also demonstrated a higher rate of wound complications in patients undergoing open abdominal surgery. In Chan’s study, this proportion was 20%, while in our study it was 53%.^10^ These complications were not observed with the laparoscopic surgical approach, demonstrating the significant benefit of this technique for the postoperative recovery of these patients.

Although Cheng and Urusank *et al*.^5,10^ similarly reported fewer postoperative complications with laparoscopic surgery, our study differs in the method used to record complications. In our research, to increase objectivity, we classified postoperative complications using the internationally recognized Clavien-Dindo system. We found a statistically higher proportion of Grade II and III complications in patients operated on via open abdominal approach. Grade II complications in the Clavien-Dindo system include events requiring therapeutic interventions beyond those allowed for Grade I complications (antiemetics, antipyretics, analgesics, diuretics, electrolytes, and physiotherapy). This also involves complications that require blood transfusions or total parenteral nutrition.^13^ It should be noted that, based on chart review, it was sometimes difficult to determine whether a patient experienced a normal postoperative course or one that would be classified as Grade I. For example, if a patient received antibiotics postoperatively, it is unclear from the records whether these were administered prophylactically or therapeutically. Therefore, a markedly higher number of laparoscopic patients were classified as Grade I or as having a normal postoperative course compared to the open abdominal group.

Our study primarily focused on comparing complications associated with two different surgical approaches for treating morbidly obese patients with endometrial carcinoma. In future research, it would also be valuable to compare treatment outcomes, including five-year recurrence rates and overall five-year survival rates.

Based on our findings, it can be concluded that, in our setting, the laparoscopic approach offers advantages over open abdominal surgery for morbidly obese patients whenever technically feasible. Assuming sufficient laparoscopic expertise, all surgeries for morbidly obese patients with endometrial carcinoma should initially be approached laparoscopically. In cases of challenging anatomy, conversion to open abdominal surgery should be performed to complete the planned procedure. As demonstrated in our study, this approach significantly reduces hospital stay, postoperative anemia, the need for transfusion, the number of reoperations, and wound-related complications.

In recent years robotic-assisted surgery has increasingly become the preferred minimally invasive approach in many centers, as available evidence demonstrates comparable oncologic outcomes to open surgery while offering clear technical advantages in high-BMI patients, including improved ergonomics, enhanced visualization, and greater precision, which facilitate complex pelvic procedures and reduce conversion rates.^24,25^

## Acknowledgments

The authors would like to acknowledge the valuable contributions of the surgeons involved in the management of some of the cases described in this report.
